# Supplementary material for: On the Origin of the Blue Color in The Iodine/Iodide/Starch Supramolecular Complex
Source: Molecules. 2022 Dec 16;27(24):8974. doi: 10.3390/molecules27248974 (PMC9784209; doi:10.3390/molecules27248974)
Supplement: Supplementary file 1 [file molecules-27-08974-s001.zip › molecules-2075125-supplementary.pdf]

Supporting Information

On the origin of the blue color in the iodine / iodide / starch supramolecular complex

Szilárd Pesek, Maria Lehene, Adrian M.V. Brânzanic, Radu Silaghi-Dumitrescu

Department of Chemistry, Babeș-Bolyai University, 11 Arany Janos str, Cluj-Napoca 400028, Romania

Iodine experiments

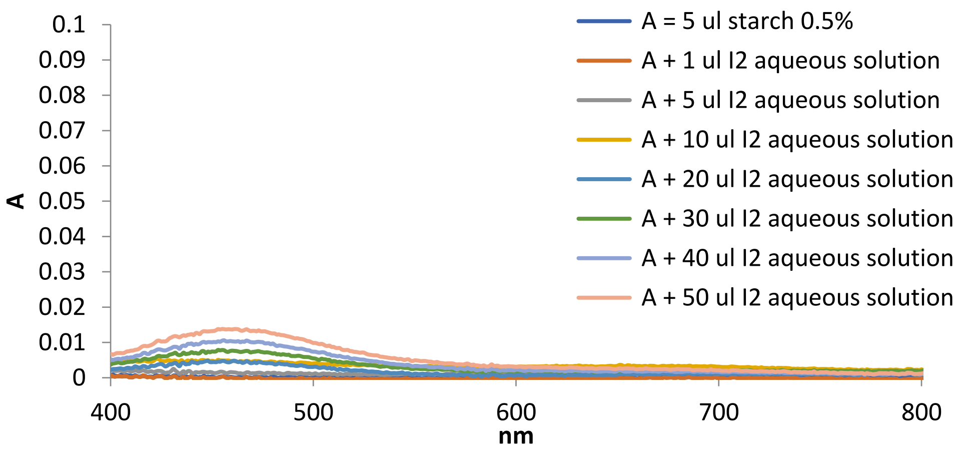

Figure S1. UV-vis spectra of starch – I_2 mixtures. Conditions: 5 µL starch 0.5%, to which indicated amounts of I_2 aqueous solution were added.

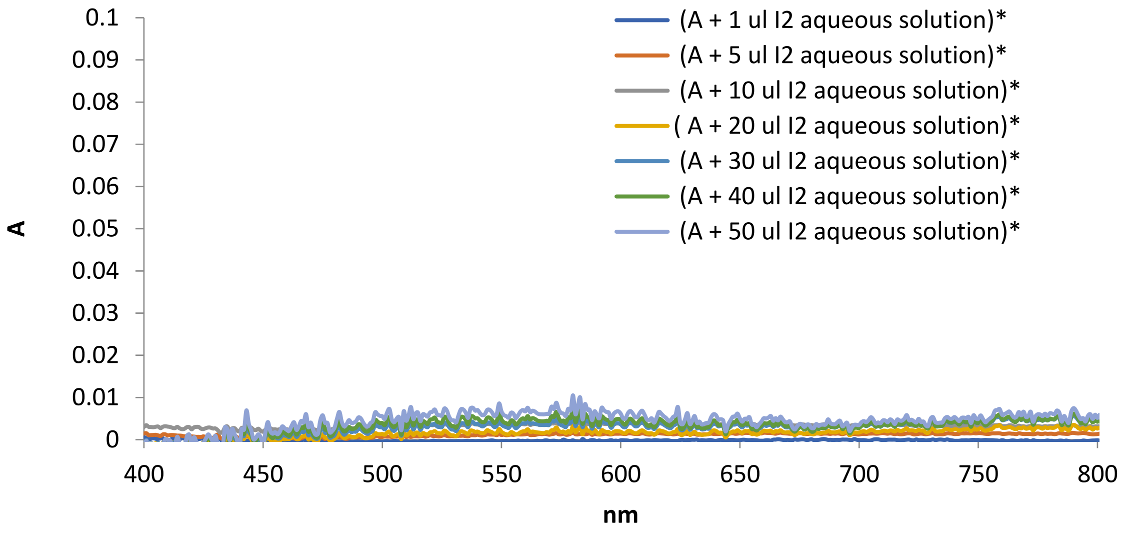

Figure S2. Same data as in Figure S1, after subtracting the from each spectrum the corresponding spectrum of a starch-free I_2 aqueous solution of the same concentration.

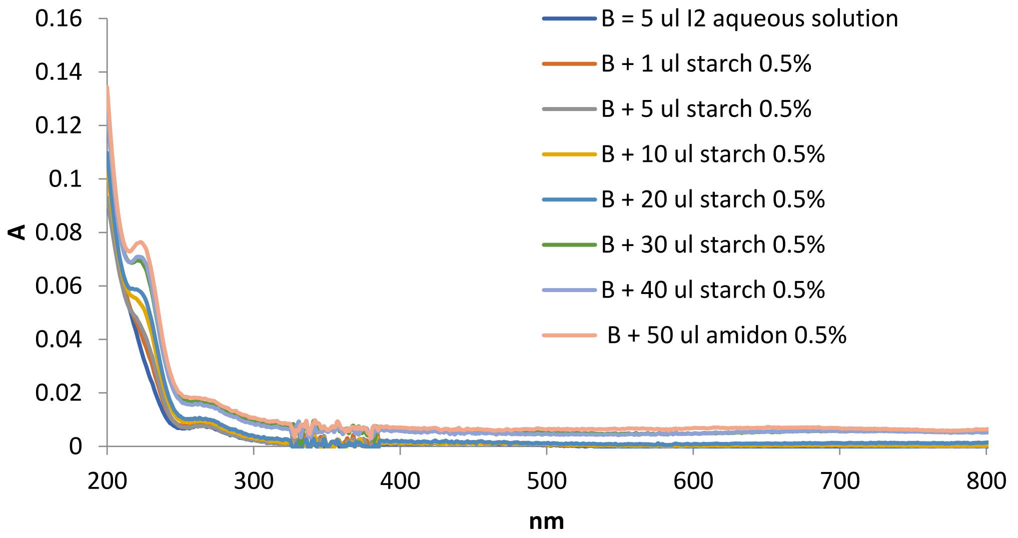

Figure S3. UV-vis spectra of an I_2 aqueous solution, to which indicated amounts of starch 0.5% solution were added.

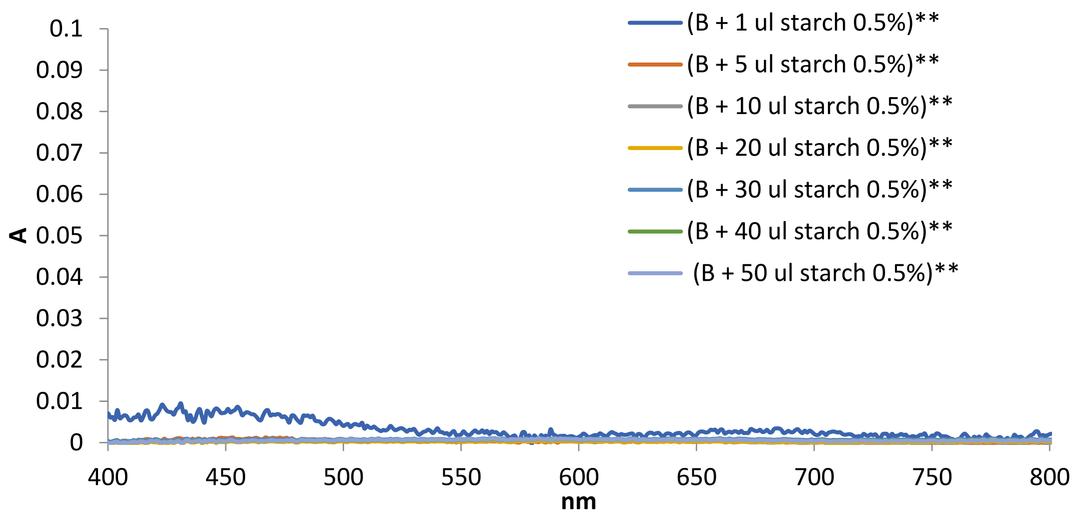

Figure S4. Corrections made to spectra from Figure S3 by subtracting the control spectra of starch at indicated concentrations.

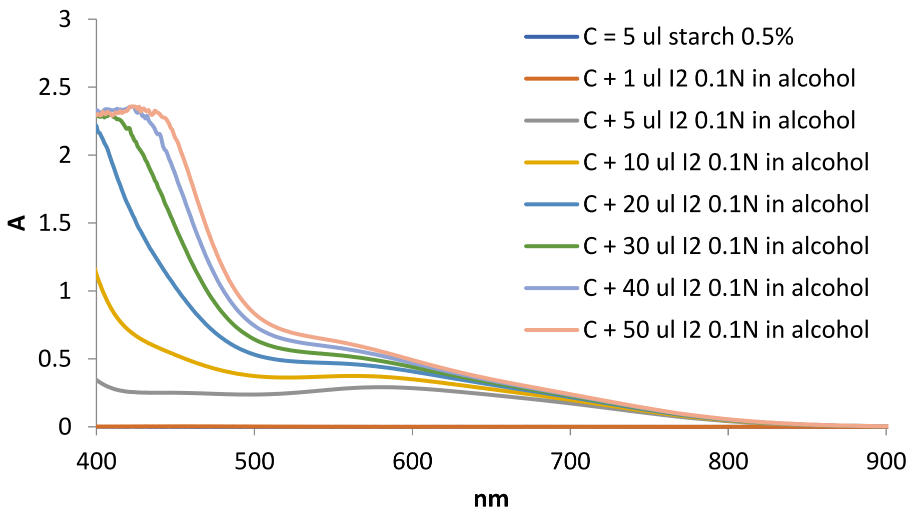

Figure S5. UV-vis spectra of starch 0.5%, to which different amounts of solution of I_2 0.1N in alcohol were added.

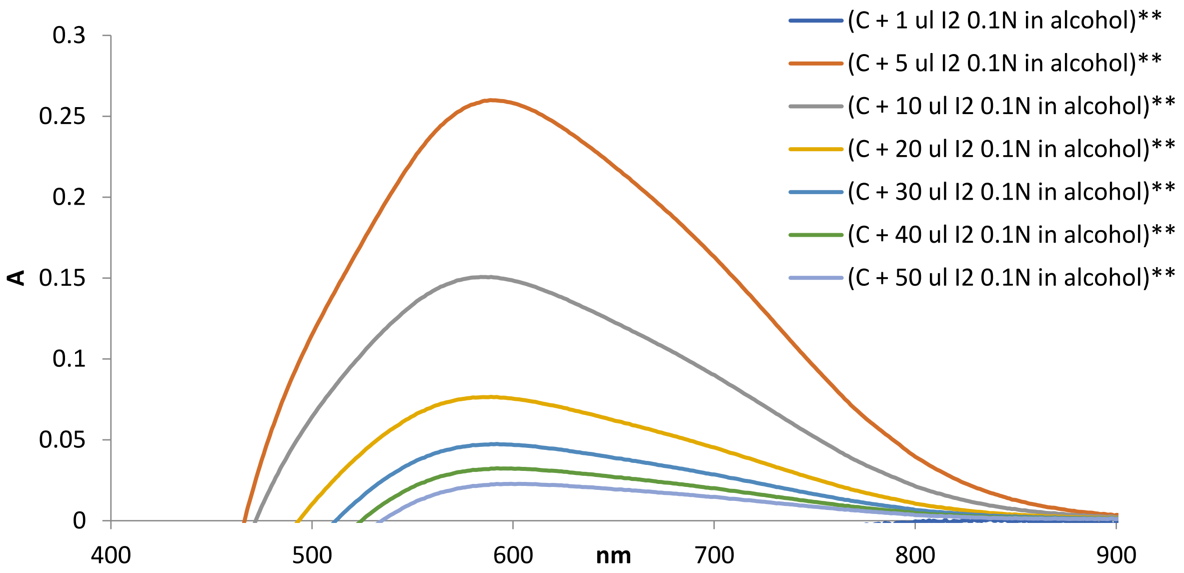

Figure S6. Corrections made to spectra from Figure S5 by subtracting the control spectra of I_2 at indicated concentrations.

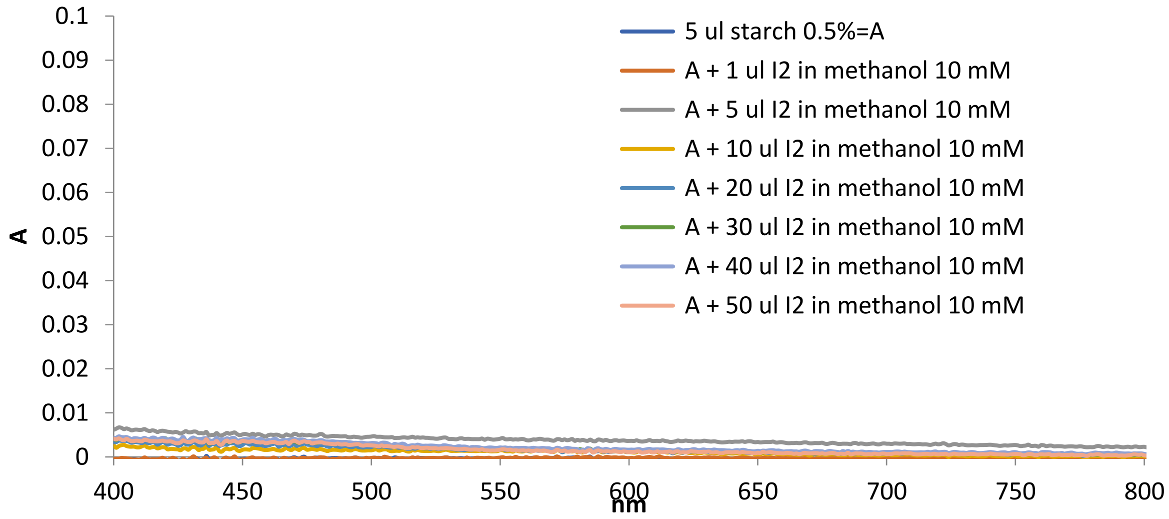

Figure S7. UV-vis spectra of starch 0.5%, to which indicated amounts of solution I_2 in methanol 10 mM were added.

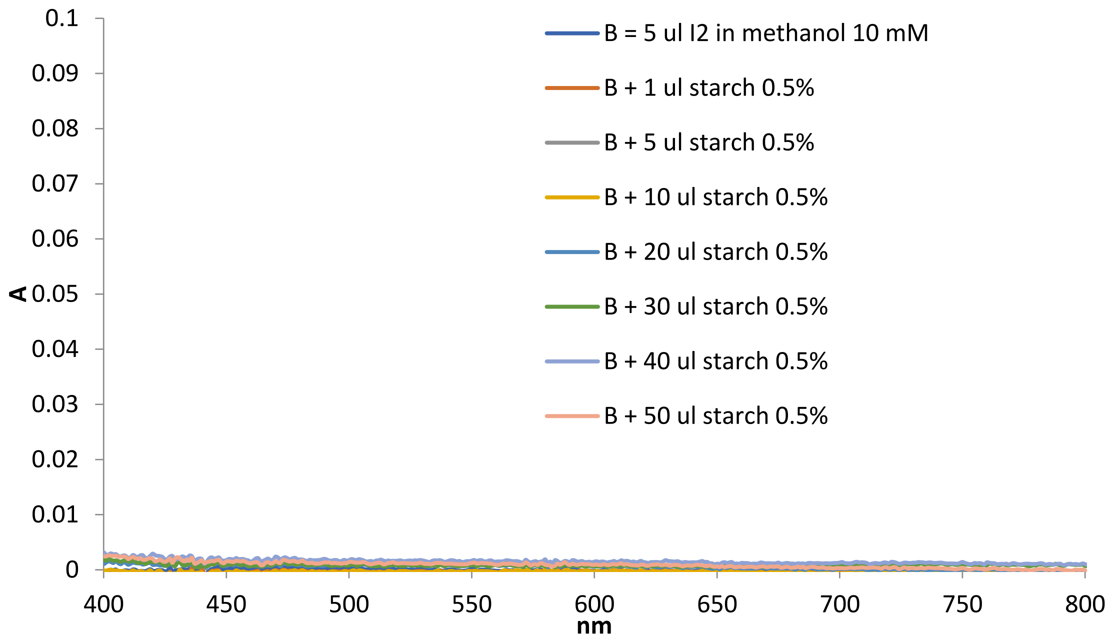

Figure S8. UV-vis spectra of water, to which I_2 from a 10 mM stock solution was added, followed by indicated amounts of 0.5% starch solution.

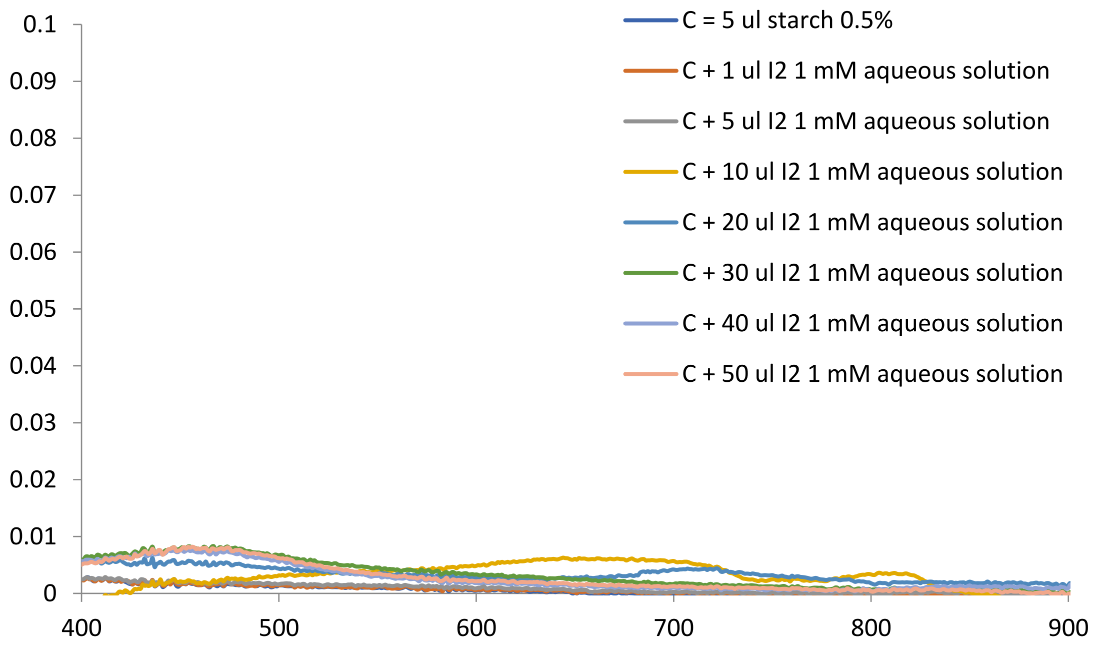

Figure S9. UV-vis spectra of starch solutions to which indicated amounts of I_2 1 mM aqueous solution were added.

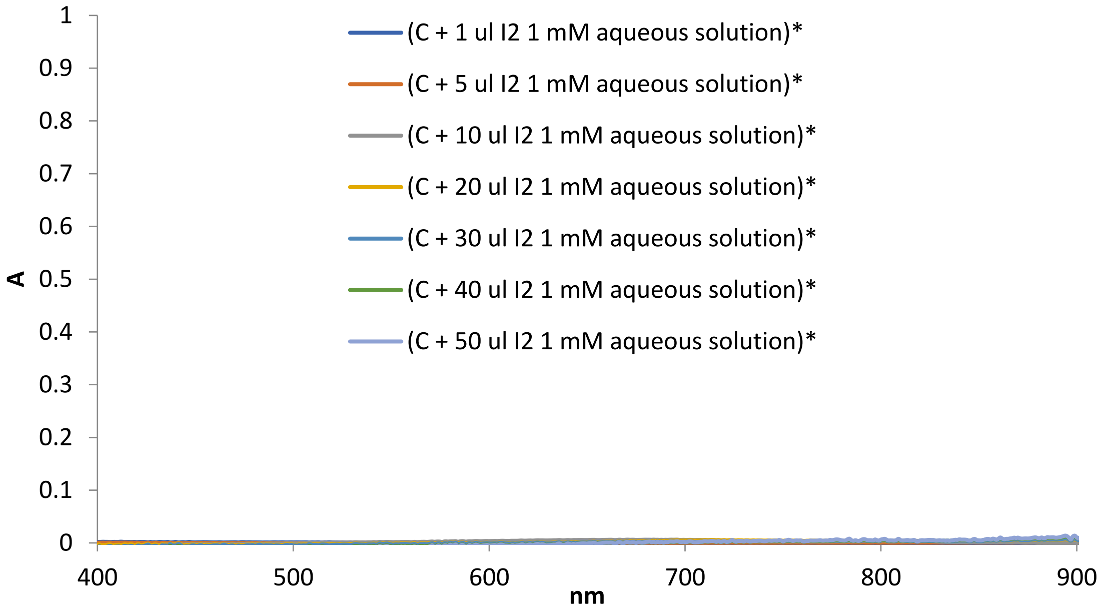

Figure S10. Corrections made to spectra from Figure S9 by subtracting the control spectra of I_2 at indicated concentrations.

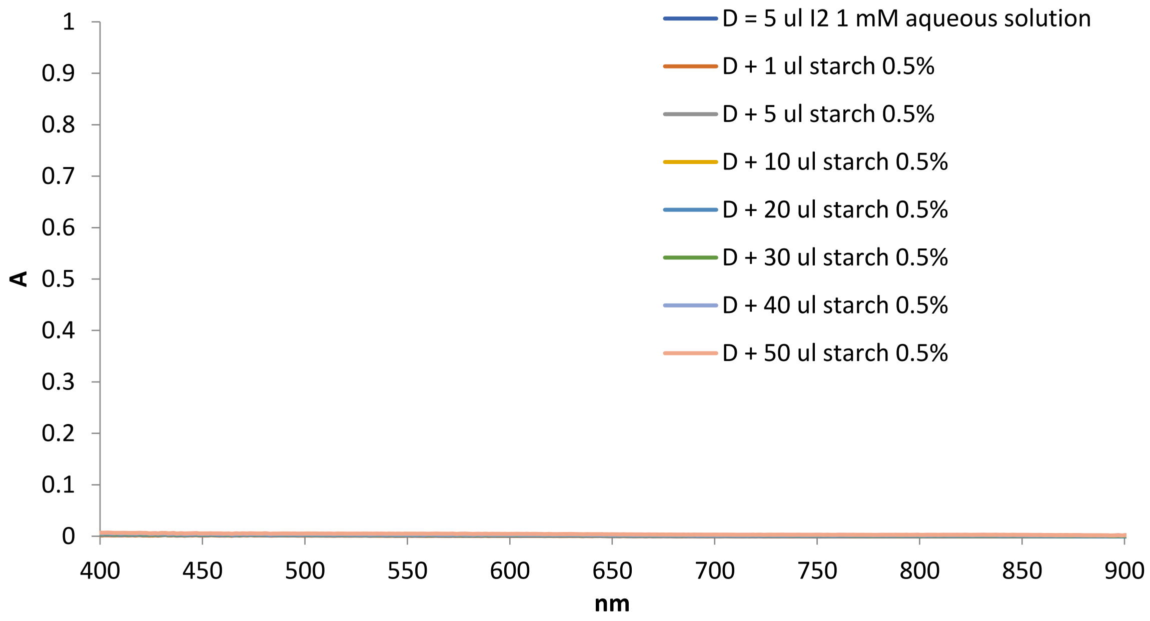

Figure S11. UV-vis spectra of a 1 mM I_2 aqueous solution, to which indicated amounts of starch 0.5% solution were added.

Experiments with iodine-iodide

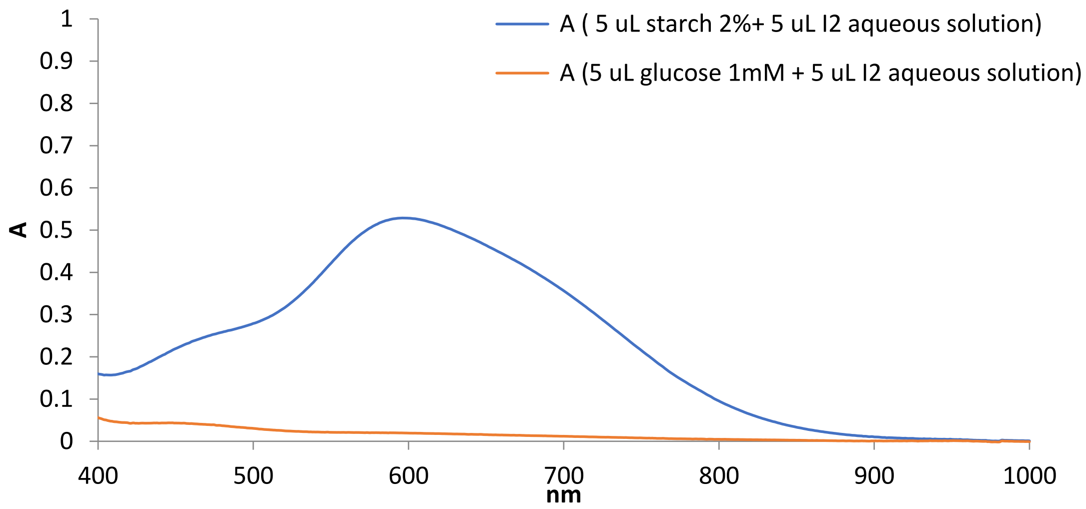

Figure S12. UV-vis spectra of starch 2% / glucose 1 mM to which 5 μL solution of iodine-iodide aqueous solution were added.

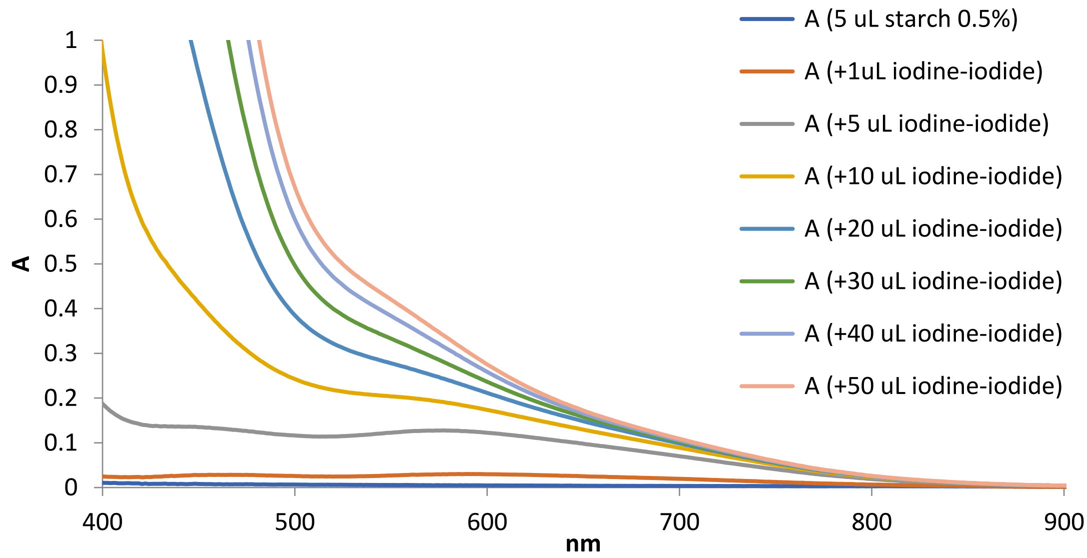

Figure S13. UV-vis spectra of starch 0.5%, to which iodine-iodide was added.

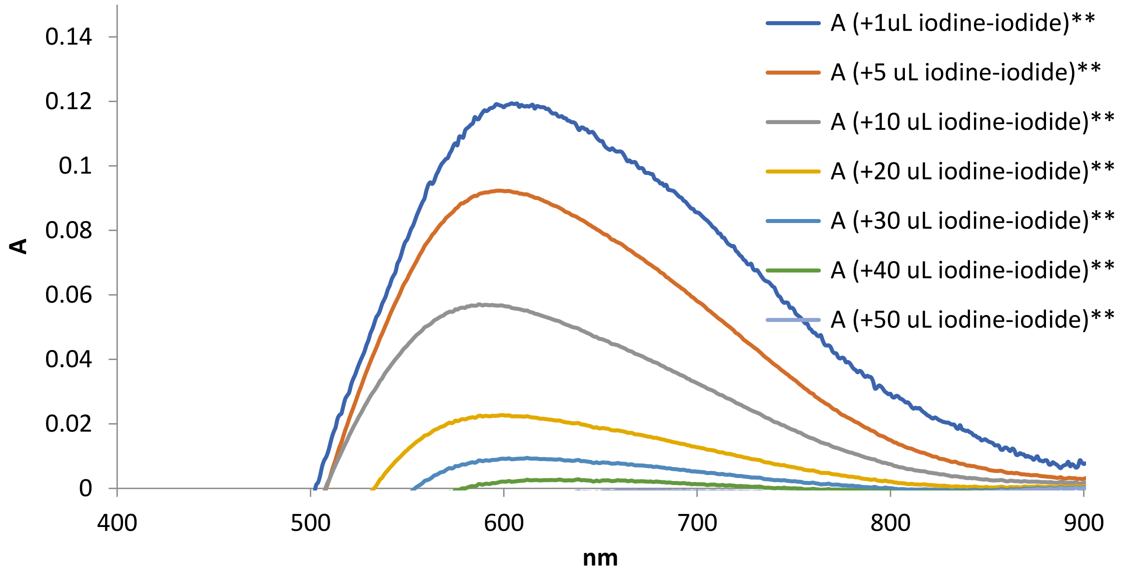

Figure S14. Same spectra as in Figure S13, now corrected by subtracting the control spectra of iodine-iodide solutions collected in the absence of starch.

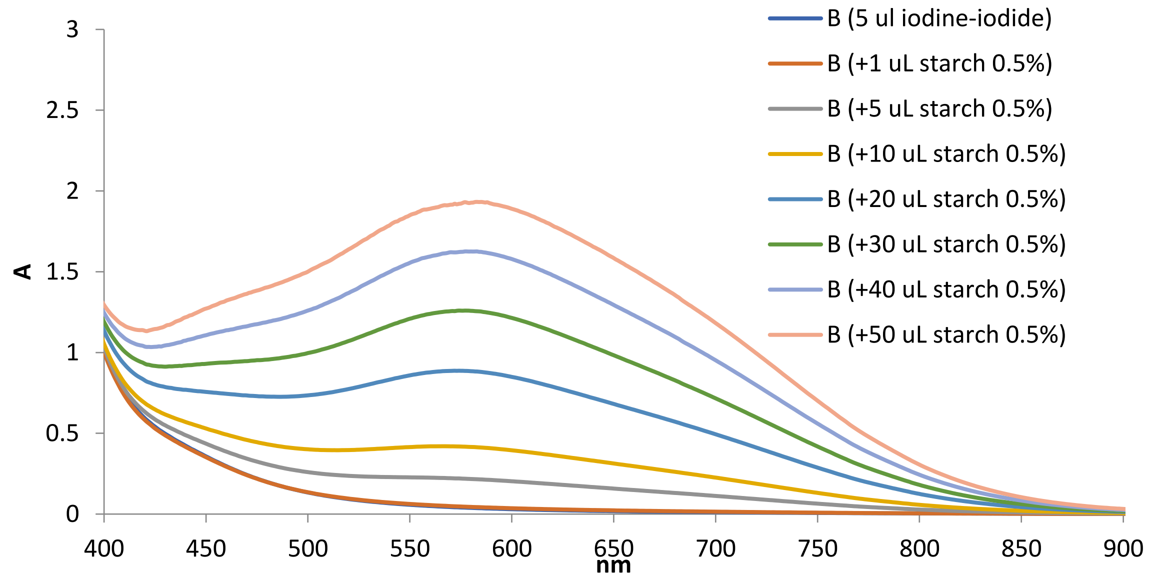

Figure S15. – UV-vis spectra of an iodine-iodide aqueous solution to which indicated amounts of starch 0.5% solution were added.

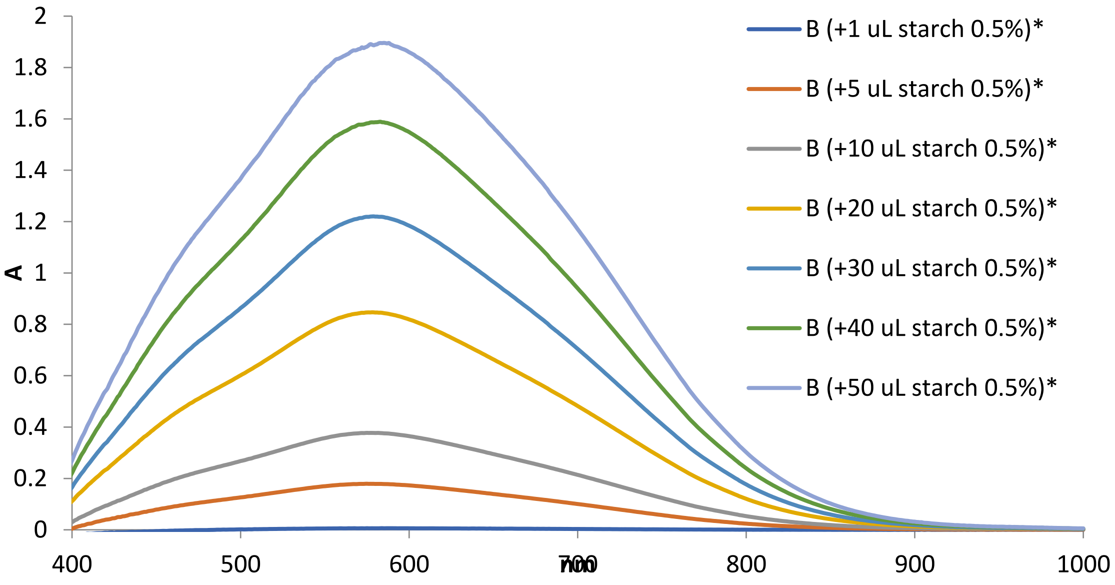

Figure S16. Same spectra as in Figure S15, now corrected by subtracting the control spectra of starch solutions collected in the absence of iodine.

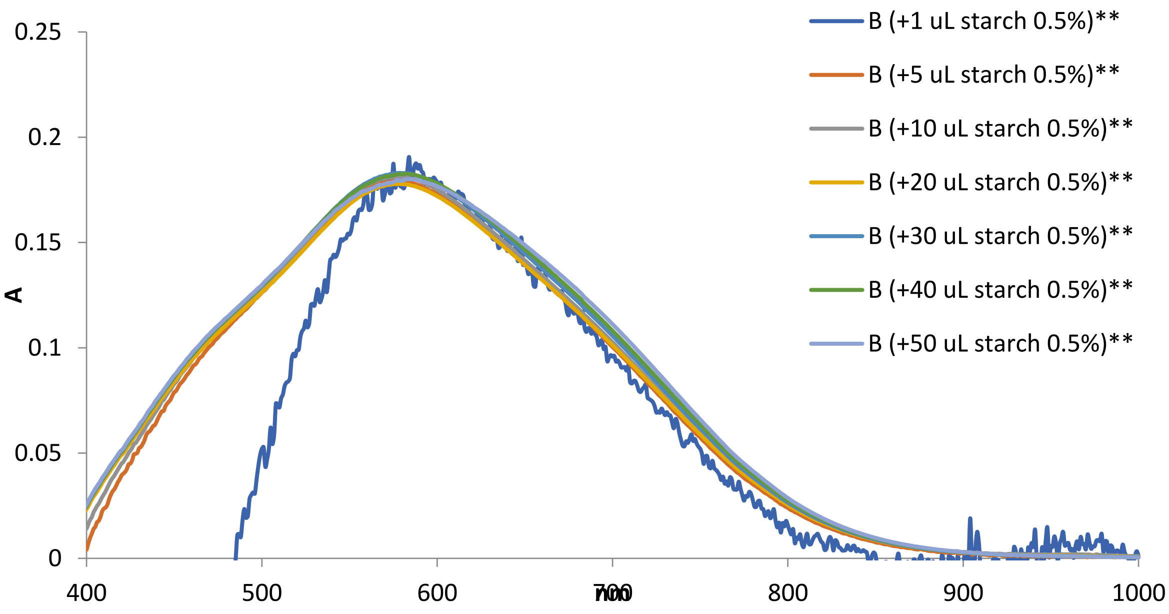

Figure S17. Same spectra as in Figure 16, multiplied by arbitrary constants each, so that a clearer comparison of the shapes of the maxima can be offered.

Experiments with amylase

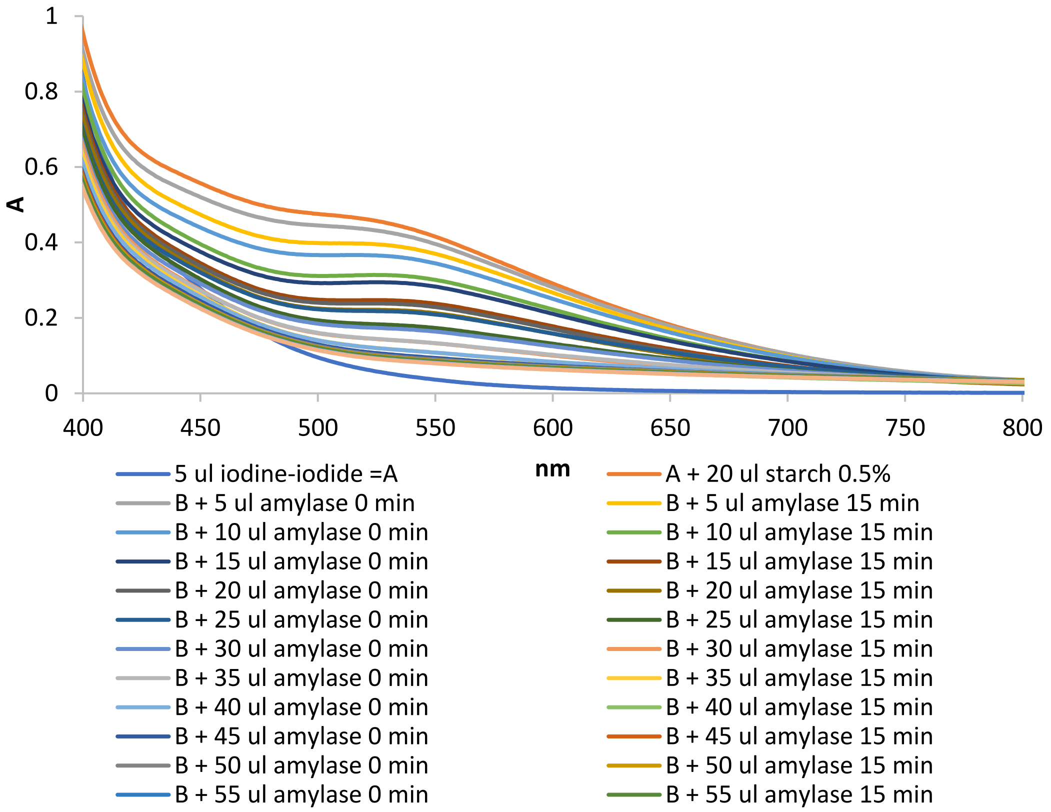

Figure S18. Time evolution of UV-vis spectra of an iodine-iodide mixture, to which starch and amylase were added.

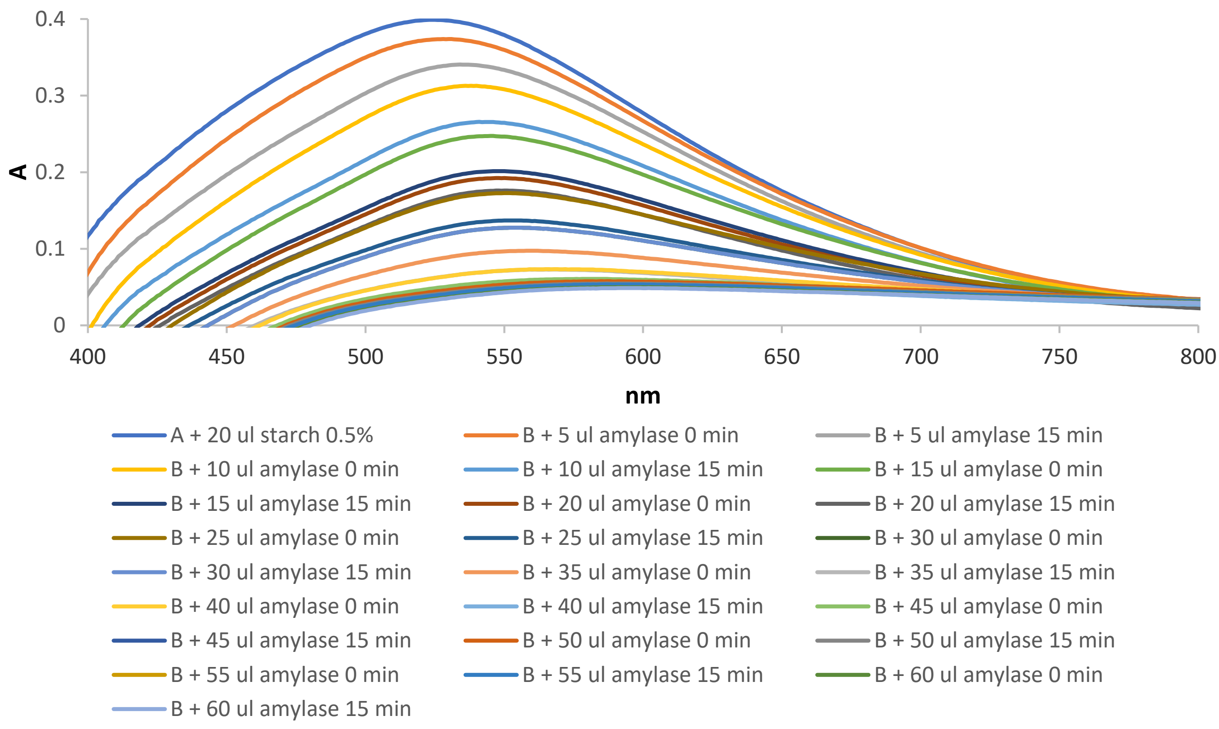

Figure S19. Same spectra as in Figure S18, now corrected by subtracting the control spectrum of the iodine-iodide solution.

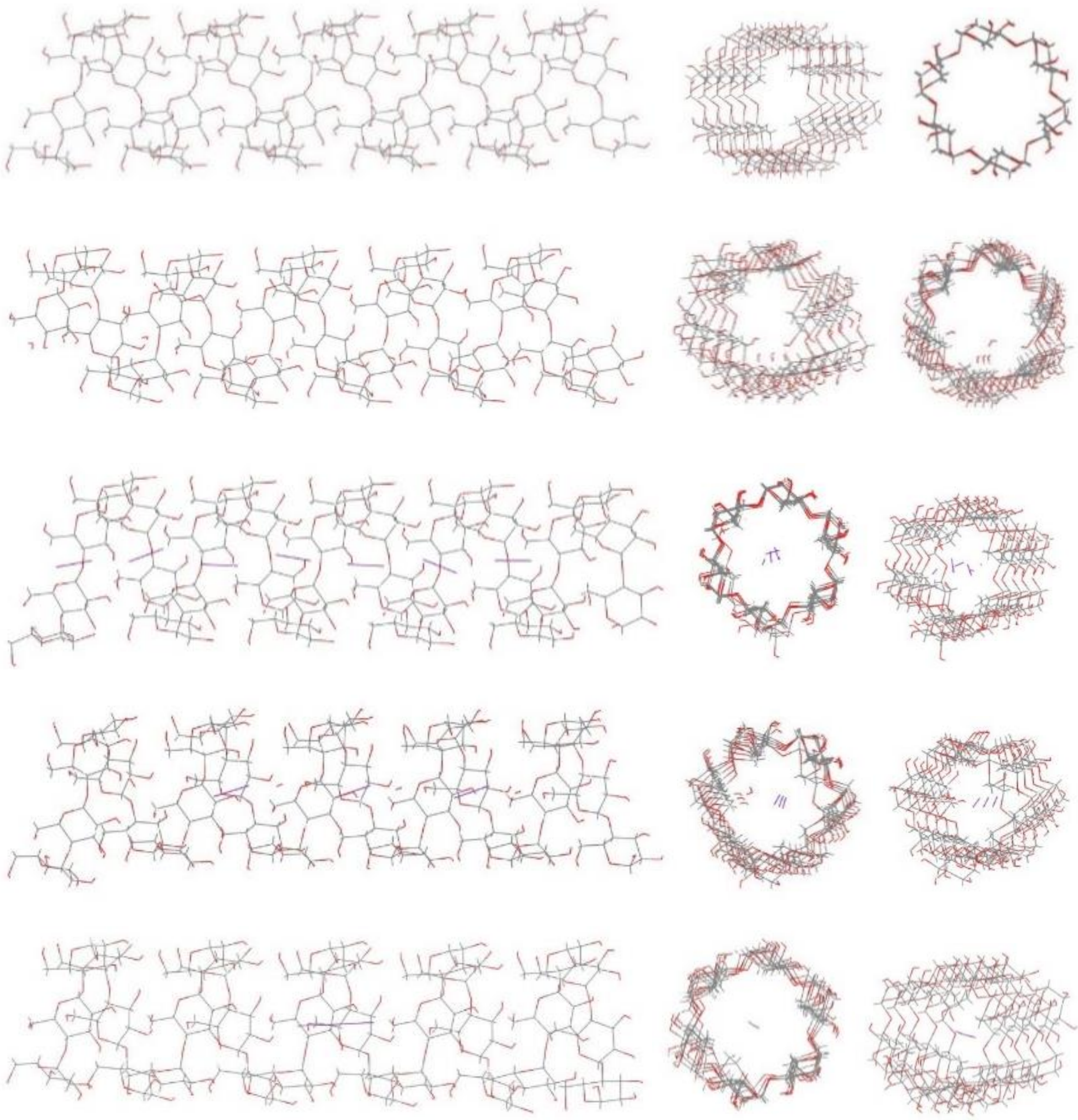

Figure S20. - Structures of AM1-optimized amylose models, in order of rows: A, A-H_2O, A-I_2, A-I_2-H_2O, A-I_3^-.

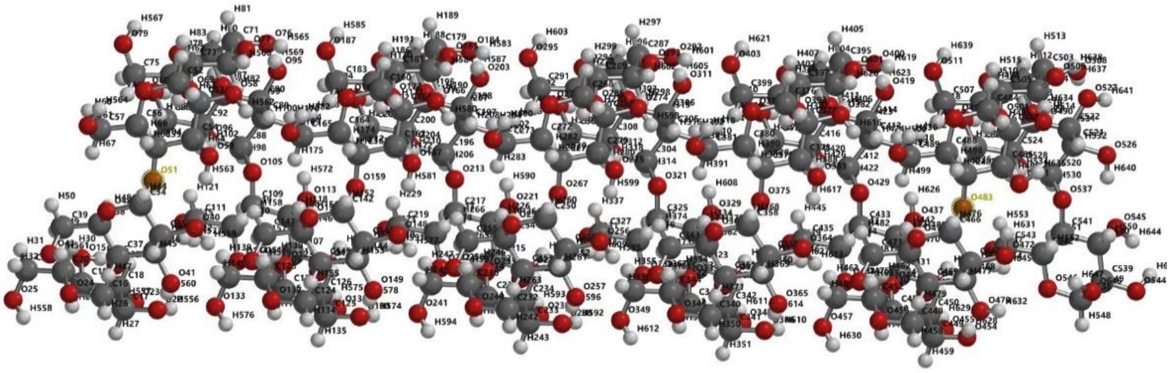

O51-O483

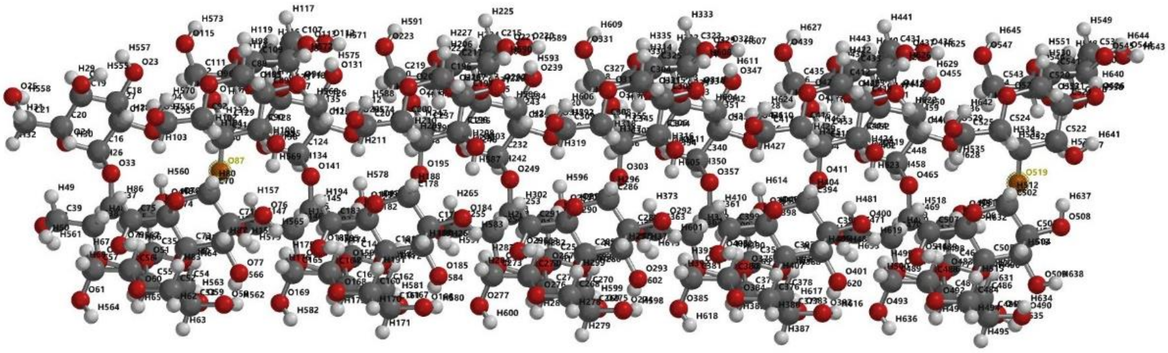

O87-O519

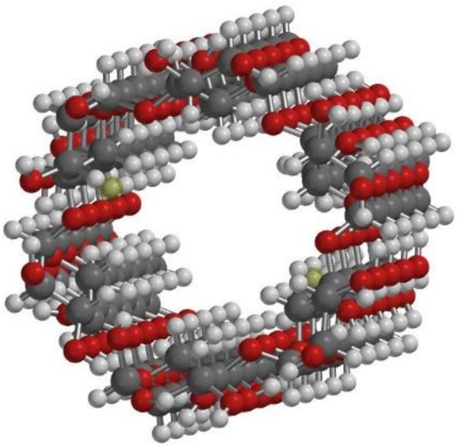

H424-H481

Figure S21. Structure of the amylose model helix, showing in yellow highlight the atoms employed for estimating the changes in helix length/diameter (as discussed in main text, Table 1).

Table S1. Key distances (Å) derived computed for iodine-amylose models. The starting geometry of the A models featured a length of 32.69 Å (measured from to O51 to O483 and O87 to O519) and a diameter of 7.04 Å (measured from to H424 to H481, cf. Supporting Information).

**Model**	**Helix length**	**Helix diameter**	**I-I distances**
I_2	-	-	2.54
I_3^-	-	-	2.62
$(I_2)_3$	-	-	Intramolecular:2.55 / 2.55 / 2.55<br>Intermolecular: 2.94 / 2.94
A	32.90	7.15	-
A-H_2O	34.38	7.82	-
A-I_2	33.08	7.44	Intramolecular: 2.55 / 2.56 / 2.56 / 2.56 / 2.56 / 2.56 / 2.55<br>Intermolecular: 2.85 / 2.84 / 2.84 / 2.83 / 2.83 / 2.84
A-I_2-H_2O	34.00	7.89	Intramolecular: 2.65 / 2.65 /2.65<br>Inter-I_2: 7.60 / 5.80<br>H_2O-I: 4.56 / 5.09 / 3.92 / 3.92 / 3.79 / 3.93
A-I_3^-	34.04	7.14	2.65 / 2.65

Table S2. AM1 energies (kcal/mol) of amylose models employed in the present study.

	**Energy**	**Total interaction energy**	**Interaction energy per small molecule**
H_2O	-59.2	-	-
I_2	19.8	-	-
I_3^-	-42.3	-	-
$(I_2)_3$	49.4	-10.0	-3.3
A	-7224.1	-	-
A-H_2O	-7547.8	-27.7	-5.5
A-I_2	-7083.0	2.5	0.4
A-I_2-H_2O	-7406.2	-4.7	-0.7
A-I_3^-	-7275.6	-9.2	-9.2

Table S3. Solvent effects on TD-DFT-predicted maxima and oscillator strengths for selected iodine/iodide models.

Model	ε (solvent)	Wavelength (nm)	Oscillator strength
I_2	1.0 (vacuum)	588	0.0015
I_2	9.1 (dichloromethane)	608	0.0014
$(I_2)_2$	1.0 (vacuum)	692	0.0019
$(I_2)_2$	9.1 (dichloromethane)	646	0.0019
I_3^-	1.0 (vacuum)	372	0.0000
I_3^-	9.1 (dichloromethane)	365	0.0000
I_5^-	1.0 (vacuum)	365	0.0007
I_5^-	9.1 (dichloromethane)	360	0.0007
I_2--I_3^---I_2	1.0 (vacuum)	680	1.5507
I_2--I_3^---I_2	2.3 (benzene)	648	1.5668
I_2--I_3^---I_2	9.1 (dichloromethane)	566	1.4483
I_2--I_3^---I_2	32.7 (methanol)	541	1.4483
I_2--I_3^---I_2	80.0 (water)	537	1.3979
I_2—I_5^---I_2	1.0 (vacuum)	681	1.9297
I_2—I_5^---I_2	9.1 (dichloromethane)	548	2.2216
I_2—I_5^---I_2	32.7 (methanol)	527	2.1917
